# Impact of Intermittent Fasting Combined With High-Intensity Interval Training on Body Composition, Metabolic Biomarkers, and Physical Fitness in Women With Obesity

**DOI:** 10.3389/fnut.2022.884305

**Published:** 2022-05-26

**Authors:** Gabriela Batitucci, Eli V. Faria Junior, Jonatas E. Nogueira, Camila F. C. Brandão, Gabriela F. Abud, Gabriela U. Ortiz, Julio S. Marchini, Ellen C. Freitas

**Affiliations:** ^1^Department of Food and Nutrition, School of Pharmaceutical Sciences of Araraquara, State University of São Paulo - FCFAR/UNESP, Araraquara, Brazil; ^2^Department of Health Sciences, Ribeirão Preto Medical School, University of São Paulo - FMRP/USP, Ribeirão Preto, Brazil; ^3^Laboratory of Exercise Physiology and Metabolism, School of Physical Education and Sports of Ribeirão Preto, University of São Paulo - EEFERP/USP, Ribeirão Preto, Brazil; ^4^Internal Medicine Department, Ribeirão Preto Medical School, University of São Paulo, Ribeirão Preto, Brazil

**Keywords:** obesity, intermittent fasting (IF), high-intensity interval training (HIIT), fat-free mass, nitrogen balance

## Abstract

**Background:**

Intermittent fasting (IF) is a dietary approach that is widely popular due to its effects on weight and body fat loss, but it does not appear to ensure muscle mass preservation. Incorporating high-intensity interval training (HIIT) into an individual’s routine could be an attractive and viable therapeutic option for improving body composition, lifestyle and health promotion. Problematizing the emerging situation of fighting obesity, led us to clarify gaps about IF and hypothesize that IF and HIIT in conjunction may protect against muscle mass decline without impairing nitrogen balance (NB), in addition to improving the physical fitness of women with obesity.

**Objectives:**

To evaluate the effects of IF alone and combined with HIIT on body composition, NB and strength and physical fitness in women with obesity.

**Methods:**

Thirty-six women (BMI 34.0 ± 3.2; 32.2 ± 4.4 years) participated and were randomly distributed into three groups: (1) Intermittent fasting combined with exercise group (IF + EX); (2) Exercise group (EX); and (3) Intermittent fasting group (IF). The interventions took place over 8 weeks and all evaluations were performed pre and post-intervention. The HIIT circuit was performed 3x/week, for 25 mins/session, at 70–85% of the maximum heart rate. The intermittent fasting protocol was a 5:2 diet with two meals within 6 h on fasting days, being 25% of total energy intake, plus 18 h of complete fasting. The protocol was performed 2x/week and 5 days of *ad libitum* ingestion. Resting metabolic rate (RMR) was measured by indirect calorimetry, body composition by BodPod^®^, NB from urinary nitrogen, food consumption by food records and physical and strength performance were measured by physical tests. ANOVA two-way repeated measures mixed model was performed followed by Sidak *post hoc* (*p* < 0.05). This project was registered in ClinicalTrials.gov, NCT05237154.

**Results:**

There were a reduction in body weight (*P* = 0.012) and BMI (*P* = 0.031) only in the IF + EX group. There was body fat loss in the IF + EX group (−4%, *P* < 0.001) and in the EX group (−2.3%, *P* = 0.043), an increase in fat-free mass in the IF + EX group (+3.3%, *P* < 0.001) and also in the EX group (+2%, *P* = 0.043), without differences between groups and the IF group showed no changes. The NB was equilibrium in all groups. All parameters of aerobic capacity and strength improved.

**Conclusion:**

Combining IF with HIIT can promote increments in fat-free mass, NB equilibrium and improve physical fitness and strength.

## Graphical Abstract

HIIT, high-intensity interval training; IF, intermittent fasting. The HIIT training protocol developed in the present study was efficient in improving health and lifestyle parameters of women with obesity, but combining the 5:2 intermittent fasting protocol with HIIT training seems to further enhance these advantages, promoting more benefits to body composition, fat-free mass gain, and improved strength and aerobic capabilities. Performing IF in isolation, without the participation of exercise, may not be a good strategy for changes in body composition and energy metabolism. Given the importance of maintaining a positive nitrogen balance, both HIIT and IF promoted nitrogen balance equilibrium. 
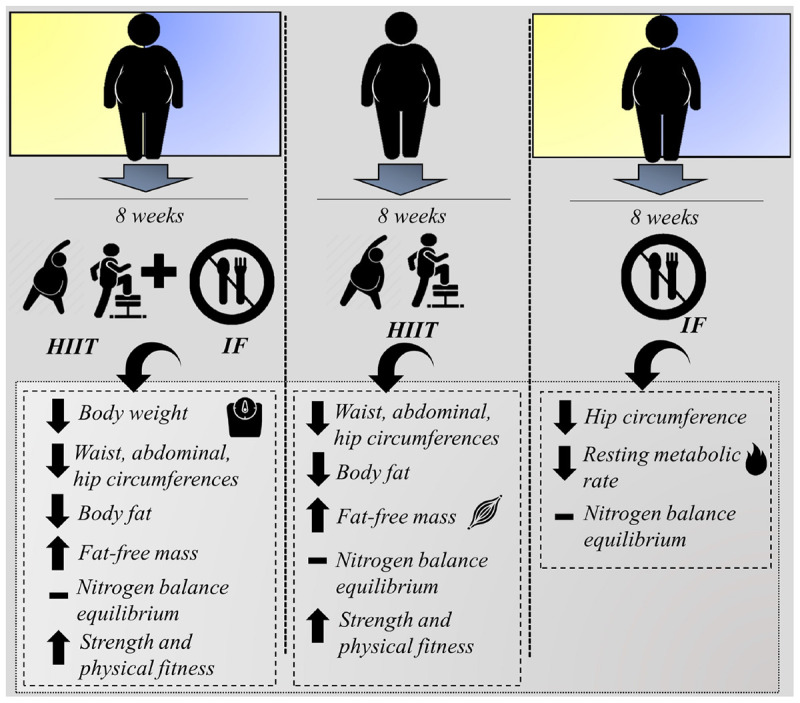

## Introduction

The prevalence of obesity affects more than 2 billion people worldwide, especially women who are most affected by this chronic condition (1). Given this scenario, the impact of obesity on clinical and public health perspectives represents an emergency, with increased risks of cardiovascular disease, diabetes, cancer, and osteoarthritis (2, 3). Obesity is a pathophysiological condition of predominantly multifactorial etiology, in which the “state of the art” continues to be the search for efficient therapeutic strategies for the treatment and prevention of this underlying disease (4). The majority of therapies involve changes in eating habits and non-sedentary lifestyle, thus intermittent fasting (IF) and high-intensity interval training (HIIT) have gained prominence in the scientific community as new approaches to the management of obesity, demonstrating effects on weight loss and body fat and on cardiometabolic health in obesity (5–8).

The synergistic effects of IF and HIIT are still poorly explored, especially in humans, once that IF when not combined with exercise seems to culminate on body fat reduction, but subtly, without effects on skeletal muscle (9). In this sense, a recent clinical trial observed discreet results in weight and body fat loss in overweight and obese individuals undergoing IF, but variables of fat-free mass and physical fitness were not evaluated (10).

The fact is that restrictive diets, such as IF, with a focus on weight loss can lead to a reduction in body fat (7), but also to unintentional decline in muscle mass, which means an unproblematic condition in the metabolic view, including energy, glucose and insulin metabolism (11, 12). However, there is an aggravating factor when found a disproportionate and unhealthy loss of muscle mass, which can then result in metabolic impairment, especially in protein metabolism (12).

Muscle mass is an important metabolic health predictor and, in that perspective, it is essential to ensure the maintenance or increase in muscle mass during the process of caloric restriction induced by diets (13). In this context, different outcomes in body composition have been observed with IF, setting precedents for the advancement of new studies that accurately elucidate the effects of the different protocols used (14). The Alternate Day Fasting (ADF) protocol, which consists of 24-h fasting, has already been shown to maintain lean mass (15) or deplete fat-free mass (9) in the absence of physical exercise. Using the IF strategy in obesity appears to have a greater ability to retain muscle mass, as opposed to continuous caloric restriction (16). However, there are few studies investigating the impact of the 5:2 intervention on fat-free mass, as current discussions strongly emphasize the effects on weight and body fat loss and the study by Patikorn et al. (17) fortify this idea by suggest that 5:2 intermittent fasting protocol applied for a long period can result in weight and fat mass loss in overweight or obese adults in a way that is superior to time-restricted eating or ADF (17).

Furthermore, another relevant aspect involving the different IF protocols is linked to the distribution of macronutrients in the period of caloric deficit, considering that, especially, the protein supply strongly affects fat-free mass and nitrogen balance (NB) (18). The NB is an important marker of depletion or increment the fat-free mass (19) and is intimately related to the individual’s protein consumption in 24 h (20). Hypocaloric strategies without adequate protein intake can lead to a negative NB and drive muscle mass depletion processes. On the other hand, approaching a positive or “*equilibrium*” NB is important for muscle gain or maintenance, and it is essential to ensure that protein consumption is not mitigated (21). Clinical trials with ADF protocols which guaranteed a protein supply observed maintenance of fat-free mass (15). In that regard, protein supply during energy deficit in IF is still controversial, ranging from 15 to 35% (15, 22). We prioritized offering 40% protein sources, as dietary protein plays an important role in obesity management acting on satiety mechanisms (23), may corroborate with a better tolerance to periods of fasting.

High-intensity interval training has been presented as an efficient strategy to promote the reduction of body fat, even in the absence of weight loss. In addition, high-intensity and short-duration training has proved to be a more attractive option for the sedentary population with overweight and obesity, compared to other modalities (8). Compared to continuous moderate-intensity exercise, HIIT combined with resistance exercise has been shown to improve aerobic capacity and strength in obese individuals, in addition to conferring advantages in attenuating muscle mass decline (8, 19).

Therefore, combining IF with high-intensity exercise may be a promising strategy for mitigating the risks of unhealthy lean mass loss (22), as well as improving the functional physical capacities of sedentary individuals with obesity during the weight loss process (13).

Thus, the present investigation hypothesizes that the combination of IF and HIIT would preserve fat-free mass, not affect nitrogen balance, and improve physical fitness in women with obesity. Based on the aforementioned, the present study evaluated the effects of IF alone and combined with HIIT on body composition, NB and parameters of physical fitness and strength.

## Materials and Methods

### Study Design and Subjects

A randomized study was conducted. Participants were recruited through adverts on social networks. Thirty-six women with obesity degree one according to their body mass index (BMI 34.0 ± 3.2; 32.2 ± 4.4 years old) participated in this study. Exclusion criteria were: having a diagnosis of diabetes, hypertension, dyslipidemia, heart disease, osteoarthritis, gastritis; smokers; alcoholics; using regular medications; who were undergoing weight loss treatment; lack of availability for training and meetings; who reported malaise during long periods of fasting; and being <18 years or >40 years. The Ethics Committee of the School of Physical Education and Sport of Ribeirão Preto (EEFERP-USP) and of the University Hospital of the Faculty of Medicine of Ribeirão Preto approved the study (nos. 13359319.3.0000.5659, 13359319.3.3001.5440, respectively). All subjects gave written informed consent to participate, after receiving an explanation of the purpose of the study and the risks involved. This project was registered in ClinicalTrials.gov, NCT05237154.

**FIGURE 1:**
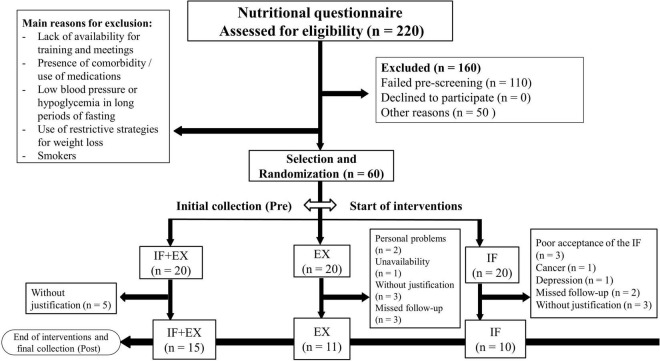
Flow diagram. IF + EX, intermittent fasting group associated with exercise; EX, exercise group; IF, intermittent fasting group.

Participants were evaluated pre and post-interventions with exercise and/or IF that lasted 8 weeks. All post-intervention assessments were performed 48 h after the last exercise session and/or the last day of the IF protocol. A random draw was performed and the participants were divided into three groups; the intermittent fasting associated with physical exercise group (IF + EX, *n* = 15), the physical exercise group (EX, *n* = 11), and the intermittent fasting group (IF, *n* = 10), totaling 36 participants. The study was widely disseminated and initially attracted a high number of individuals. However, after strictly applying the eligibility criteria, the present study started with a total of 60 participants, of which only 36 completed all stages. There was a high number of dropouts, leading in a considerable sample loss (Figure 1).

### Sample Size

For the methodological planning of the study, the sample size was calculated based on a randomized, controlled, parallel-arm study conducted by Bhutani et al. (22), who demonstrated that the combination of ADF plus exercise produces superior changes in body composition. The sample size calculation was adequate to the statistical model planned for the present study (ANOVA two-way repeated measures with mixed effects) considering the fat-free mass as a variable of primary interest. For the present study, twenty-one subjects in total (seven subjects per group) were calculated as the minimum sample to evaluate the primary outcome variables, with a mean difference of 0.66 (SD 0.58), a significance level of 0.05, and a statistical power of 80%.

### Interventions

#### Intermittent Fasting

The 5:2 intermittent fasting protocol was used, performed twice a week, on non-consecutive days, for 8-weeks of intervention (17, 24). The participants consumed 25% of the total daily energy needs on IF days (approximate total of 600 kcal that were divided into two meals), while on the other days of the week food was allowed *ad libitum*. The calculation of the average of the total energy expenditure (TEE) was performed from the average of the resting metabolic rate (RMR), obtained by indirect calorimetry, of each group that would be submitted to the protocol, multiplied by the activity factor of 1.2 for sedentary individuals (25). The IF protocol consisted of 6:18 h, with a 6 h eating period, during which participants were only allowed to consumption of two meals, followed by 18 h of complete fasting, when participants were able to ingest only water, and tea and coffee without sugar. Participants received a list of meal options duly calculated at 300 kcal each to be chosen on IF days and received the necessary counseling. The distribution of macronutrients prioritized the offer of 40% of protein sources on the menu of the IF protocol delivered to the participants. Weekly meetings were held with the groups to monitor the follow-up of the intervention. The EX-group was instructed to maintain usual eating habits throughout the intervention period. In Supplementary Material 1 are two examples of two meals consumed on the days of the IF protocol.

#### High-Intensity Interval Training

High-intensity interval training was performed on a circuit, adapted according to Sperlich et al. (19) and the training sessions were carried out at the multisport gym at the University of São Paulo (Ribeirão Preto, SP, Brazil), being fully supervised by a physical education professional. The progressive training protocol was performed 3x/week, for 8 weeks, with at least 1 day of rest between sessions and a duration of 25 mins each session, with 4 mins of initial warm-up, 18 mins for the main part, and 3 mins of relaxation (see Table 1 for additional details). In the first and second weeks, the exercise execution and recovery times were 30 s. In the third and fourth weeks, the execution time was 35 s and the recovery time was 25 s. In the fifth and sixth weeks, the execution time was 40 s and the recovery time was 20 s. In the seventh and eighth weeks, the execution time was 45 s and the recovery time was 15 s. Each session consisted of a circuit with nine exercises and the participants were required to complete two laps of the circuit. The training intensity was maintained at between 70 and 85% of the maximum heart rate (HR_max_) previously determined by a performance Shuttle Walking test. The training intensity was controlled by a cardiac monitor (Polar^®^, FT1 model) and by the rating of perceived exertion (RPE) according to Foster et al. (26).

**TABLE 1:** Physical training protocol.

Week	Session	Training
1, 3, 5, and 7	1	Two series Chest fly on suspension straps + rest
		Lateral run with sprint + rest
		Abdominal crunches + rest
		Medicine ball chest throw + rest Squats + rest
		Jackknife abdominal + rest Kettlebell swing + rest
		Suicide drills + rest
		Elevated mountain climbers + rest
	2	Two series
		Resistance band row + rest Back lunge + rest Oblique crunches + rest Battle rope + rest Jump over mini barriers with sprint + rest
		Leg raise + rest
		Elevated push-up + rest
		Changing direction drill and sprint + rest
		Star crunch + rest
	3	Two series
		Overhead medicine ball throw + rest 10-m sprint + rest
		Plank + rest Row on suspensions straps + rest
		Lunge + rest
		Oblique crunch + rest
		Resistance band high row + rest Agility ladder drill + rest
		Raised legs crunch + rest
2, 4, 6, and 8	1	Two series
		Chest fly on suspension straps + rest Forward and backward run and sprint + rest
		Crunch + rest
		Bench dip + rest Squat walking + rest
		Bicycle crunch + rest
		Resistance band front elevation + rest
		Lateral quickness drill + rest
		Leg raise + rest
	2	Two series
		Suspension strap biceps curl + rest
		Medicine ball wall throw and squat + rest
		Jackknife abdominal + rest
		Medicine ball chest throw + rest
		Forward and lateral running and sprint + rest
		Leg raise + rest
		Medicine ball biceps curl throw in air + rest
		5-m sprint + rest
		Oblique crunch + rest
	3	Two series
		Suspension strap row + rest
		Step-up + rest
		Oblique crunch + rest
		Battle rope + rest
		Lunge + rest
		Elbow to knee crunch + rest
		Run around the cone + rest
		Resistance band lateral raise + rest
		Jackknife abdominal + rest

*REST, recovery.*

### Assessments

#### Nutritional Assessment

Anthropometry was based on the measurement of weight, height using a Filizola™ electronic platform scale (Filizola SA, São Paulo, Brazil), BMI, and waist (midpoint between the last rib and the iliac crest), abdominal (abdominal region at its greatest perimeter measured over the umbilicus or 2 cm above) and hip circumferences (27). Body composition was assessed using the whole-body air displacement plethysmography method, BodPod^®^ (Life Measurement Incorporation©, Body Composition Tracking System, United States). The equipment was properly calibrated and then the participant’s body weight was determined using the BodPod^®^ weighing scale. All participants received specific clothing for the use of the equipment, including shorts, a gymnastics top, and a cap, and were instructed not to wear metallic objects during the assessment (28). All measurements were performed pre and post-intervention.

#### Dietary Intake

All participants in the three groups were instructed to complete the food record on three non-consecutive days; one day of the week that included the intermittent fasting protocol, another day of the week with *ad libitum* food, and a weekend day. The assessment of dietary intake occurred pre intervention, in the fourth week and post-intervention. Dietary data were analyzed using Dietwin^®^ software (São Paulo, Brazil) to calculate caloric intake and macronutrients.

#### Resting Metabolic Rate and Lipid Oxidation

Resting Metabolic Rate (RMR) was assessed by indirect calorimetry using QUARK-RMR equipment (COSMED, Rome, Italy) (29). The participants remained at rest for 30 mins prior to the assessment. During the evaluation, the participants remained awake, in the supine position, in a quiet room, with a temperature between 21 and 24°C, under low lighting. All measurements were performed between 8:00 and 10:00 a.m. The women were instructed to fast for 8–10 h, not to perform any physical exercise, and not to drink coffee or black tea for 24 h before the evaluation. The equipment was automatically calibrated with known gas concentrations before all evaluations, according to the manufacturer’s specifications. The volumes of oxygen consumption (VO_2_) and carbon dioxide production (VCO_2_) were measured for 20 mins. The measurements obtained in the first 5 mins were discarded to ensure that the participant reached an equilibrium state. The Weir equation (30) was used to estimate the RMR. Lipid oxidation rates were calculated according to the formulas described by Frayn (31): Lipid oxidation (g/day) = [(1.67 × VO_2_) – (1.67 × VCO_2_)] × 1,440.

#### Blood Collection and Biochemical Analyses

After 8–10 h of fasting, a sample of 4 mL of blood was collected in EDTA tubes and used to determine glycemia, total cholesterol, triglycerides, HDL-cholesterol, and LDL-c, quantified by Labtest diagnostics^®^ kits, using an enzymatic system and absorbance spectrophotometer. The calculation of the LDL-cholesterol fraction (LDL-c) was performed using the Friedewald formula: [LDL-c = (CT – HDL-c) – (TG/5)] (32).

#### Urinary Nitrogen

Samples of 5 μl of 24-h urine collection were diluted in 495 μl of hydrochloric acid (100 mM), representing a 100-fold dilution and if necessary, the solution was diluted 200, 300, 400, or even 600-fold. Next, the determination of total urinary nitrogen was performed by the pyro-chemiluminescence method (33). The measurement of urinary nitrogen associated with the results of protein intake allowed the calculation of nitrogen balance (NB) using the following equation: NB = [PTN 24 h (g)/(6.25)] – Urinary Nitrogen 24 h (g) + 4 (g) (20).

#### Physical Tests

All tests were performed pre and post-intervention. Aerobic performance was assessed by an adapted incremental Shuttle Walking test (34) and the estimated maximum oxygen consumption (VO_2_max) was determined according to Lima et al. (35). HR_max_ and RPE were recorded at the beginning, at each stage, and at the end of the test. Maximum dynamic muscle strength was assessed by the multiple maximal repetitions (RM) test in the 45° leg press and bench press. The calculation of the maximum load and the percentage were performed according to Brzycki’s equations (36). The maximum isometric muscle strength of the dorsal region and handgrip were assessed by dynamometry, using a crown dorsal dynamometer (Osvaldo Filizola, São Paulo, SP, Brazil) and a manual hydraulic dynamometer (SH5001, Saehan Corporation, Yangdeok-Dong, Masan, Korea), respectively (37). To assess the strength resistance capacity, 1-min functional tests were performed for the abdominal, free squat, and arm flexion exercises, in which the greatest number of repetitions were required to be performed in the time interval for each exercise.

In all physical tests, participants were verbally encouraged in order to achieve the best execution performance.

### Statistical Analysis

After testing the assumptions of normality by the Shapiro-Wilk test and sphericity, the ANOVA two-way repeated measures mixed model for intra and intergroup comparisons was performed, followed by the Sidak *post hoc* in cases of group × time interaction (SPSS Statistics 20^®^ software). The Wilcoxon test was applied in cases of non-parametric distribution for intra-group comparisons and the Mann-Whitney test for comparisons between groups. To characterize the subjects, the ANOVA one-way test was performed. ANOVA with Welch’s correction was performed for cases of heteroscedasticity. The results are expressed as mean ± standard deviation. The delta variation was calculated, according to the formula, Δ% = Δ(post − pre) × 100/pre. A significance level of 5% was adopted.

## Results

### Dropouts Reasons

A high number of dropouts before completing the 8 weeks of intervention was observed, being 25% in the IF + EX group, 45% in the EX group, and 50% in the IF group. We defend the idea that the main reasons that led to the high dropout rates are not supported by the low adherence to the exercise and/or IF protocol for the most part, since, in the IF + EX group we observed that 25% of the dropouts were absent from training without giving any justification, but no feedback of low adherence to the diet was reported in this group. The IF + EX group could have less tolerated the intervention period, however, the fact that this group had the dropout rate lowest does not surprise us, as we understand that physical exercise works as a motivational tool even more when combined with a nutritional intervention, especially in the obese population. In previous studies from our research group, we observed that the exercise group always had the lowest dropout rates when compared to a control group (no intervention) or another intervention. Furthermore, of the nine participants who dropped out of the intervention in the EX group, only 3 (33.3% of dropouts) dropped out due to loss to follow-up, low adherence, while the other six participants (66.6% of dropouts) did not justify dropping out of the study or had personal problems or time conflicts to attend training (unavailability). Finally, of the 10 dropouts from the IF group, 5 (50% of dropouts) had a direct relationship with the proposed protocol, with only three individuals justifying low acceptance of the diet due to side effects of the long period of fasting (such as dizziness and headaches) and two other participants justified loss to follow-up due to routine conflicts at work in consuming time-restricted food (6 h) prior to 18 h of complete fasting, but not due to low adherence to the IF. The other five participants (50% of the dropouts) presented other reasons, such as the presence of diseases or simply being absent from the study, but unrelated to the IF protocol itself. The high number of dropouts in the IF group may have had a substantial impact on the absence of effects in this group.

We highlight how challenging it is to design the best motivational strategies capable of alleviating dropout problems in randomized and well-controlled clinical trials such as in the current study.

### General Characteristics

The general characteristics of the participants are shown in Table 2. It can be observed that for some anthropometric parameters, lipid profile, and fasting glucose, the groups showed differences prior to the beginning of the interventions.

**TABLE 2:** Baseline characteristics.

	IF + EX	EX	IF	*P* Value
	(*n* = 15)	(*n* = 11)	(*n* = 10)	
Age (years)	32 ± 4.4	33 ± 3.0	30 ± 5.0	0.15
Weight (kg)	91.5 ± 8.2	84.3 ± 4.5^a^	96.4 ± 12.8	0.006
BMI (kg/m^2^)	34.0 ± 2.6	31.8 ± 2.02^b^	34.8 ± 3.2	0.037
WC (cm)	94.8 ± 7.1	97.9 ± 8.5	94.4 ± 6.5	0.49
AC (cm)	108.9 ± 5.3	108.8 ± 6.8	113.8 ± 7.6	0.25
HC (cm)	119.4 ± 6.1	118.2 ± 6.6	120.8 ± 8.1	0.7
Body fat (%)	46.1 ± 2.2	47.3 ± 3.8	50.2 ± 4.4	0.37
Fat-free mass (%)	53.8 ± 2.2	52.6 ± 3.8	49.7 ± 4.4	0.07
Total cholesterol	181.3 ± 40.0	139.2 ± 22.0^c^	172.7 ± 34.3	0.01
(mg/dL)				
LDL-C (mg/dL)	118.0 ± 34.5	79.1 ± 15.8^d^	113.0 ± 22.6	0.002
HDL-C (mg/dL)	36.1 ± 9.4	37.3 ± 3.8	29.9 ± 4.3^e^	0.04
TG (mg/dL)	135.6 ± 60.2	84.6 ± 28.2^f^	120.1 ± 36.4	0.01
Fasting glucose (mg/dL)	101.5 ± 12.5	97.4 ± 12.4	86.7 ± 7.8^g^	0.01

*BMI, body mass index; WC, waist circumference; AC, abdominal circumference; HC, hip circumference; LDL-C, low-density lipoprotein cholesterol; HDL-C, high-density lipoprotein cholesterol; TG, triglycerides; IF + EX, intermittent fasting associated with exercise group; EX, exercise group; IF, intermittent fasting group. Values expressed as mean ± standard deviation. Small letters: difference between groups (p < 0.05). ^a^EX vs IF; ^b^EX vs IF; ^c^EX vs IF + EX; ^d^EX vs IF + EX and IF; ^e^IF vs EX and IF + EX; ^f^EX vs IF + EX; ^g^IF vs IF + EX and EX, by ANOVA one-way, post hoc Sidak with Welch’s correction for LDL-C, HDL-C, and TG.*

### Anthropometry, Energy Expenditure, and Lipid Oxidation

The results of the anthropometric variables, RMR and lipid oxidation are shown in Table 3. After 8 weeks of intervention, weight loss (*p* = 0.012) and reduction in BMI (*p* = 0.031) were observed in the IF + EX group. There was a significant loss in waist, abdominal, and hip circumference measurements in the IF + EX and EX groups (*p* < 0.001) and both groups presented greater loss in waist circumference compared to the IF group post intervention (*p* = 0.008). The IF group showed a reduction only in hip circumference (*p* < 0.001). RMR decreased 8.1% in the IF group (*p* = 0.014) post-intervention, with-out changes between groups. There were no changes in intra-group and between groups in lipid oxidation.

**TABLE 3:** Anthropometric measures, resting metabolic rate and lipid oxidation pre and post intervention.

	IF + EX (*n* = 15)			EX (*n* = 11)			IF (*n* = 10)			
Variable	Pre	Post	Δ %	Pre	Post	Δ %	Pre	Post	Δ %	*p* Value
Weight (kg)	91.5 ± 8.2	90 ± 8.5*	−1.7	84.3 ± 4.5	85.5 ± 5.9	1.4	96.4 ± 12.8	95.4 ± 12.5	−1	0.012
BMI (kg/m^2^)	34 ± 2.6	33.4 ± 2.7 *	−1.7	31.8 ± 2	32.1 ± 2.2	0.85	34.8 ± 3.2	34.4 ± 3.1	−1.1	0.031
WC (cm)	94 ± 7	91 ± 7^A^	−4	97 ± 8	92 ± 7^B^	−5	94 ± 6	94 ± 5	0.3	<0.001
AC (cm)	108 ± 5	105 ± 5^C,a^	−3	108 ± 6	107 ± 5^D,a^	−2	113 ± 7	112 ± 7	−1	<0.001
HC (cm)	119 ± 6	115 ± 6^E^	−3	118 ± 6	116 ± 6^F^	−1.7	120 ± 8	119 ± 8^G^	−1.5	<0.001
RMR (kcal/day)	1.960 ± 238	1.951 ± 301	−0.25	1.988 ± 156	1.912 ± 159	−3.5	2.053 ± 400	1.849 ± 234^H^	−8.1	0.028
Lipid oxidation (g/day)	221 ± 35	228 ± 34	6	225 ± 15	230 ± 25	2	168 ± 90	185 ± 51	11	0.364

BMI, body mass index; WC, waist circumference; AC, abdominal circumference; HC, hip circumference; RMR, resting metabolic rate; IF + EX, intermittent fasting in conjunction with exercise group; EX, exercise group; IF, intermittent fasting group. Δ%: Variation between pre and post of each group. Values expressed as mean ± standard deviation. Capital letters (A–H) represent differences within the group, effect of time: ^A^post vs pre (IF + EX); ^B^post vs pre (EX); ^C^post vs pre (IF + EX); ^D^post vs pre (EX); ^E^post vs pre (IF + EX); ^F^post vs pre (EX); ^G^post vs pre (IF); ^H^post vs pre (IF). Small letter (a) represent differences between groups post-intervention (^a^IF + EX and EX vs IF); *differences within the group (p < 0.05), by ANOVA two-way repeated measures mixed model, post hoc Sidak and Welch’s correction for weight and RMR.

### Body Composition

**FIGURE 2:**
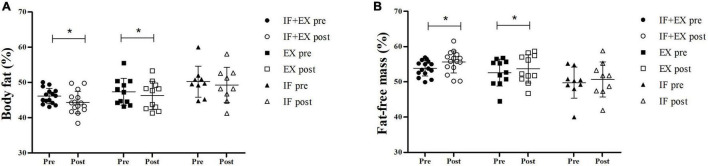
Scatter Plot to body composition pre- and post-intervention. **(A)** Body fat (%); **(B)** Fat-free mass (%). IF + EX, intermittent fasting group in conjunction with exercise (*n* = 15); EX, exercise group (*n* = 11); IF, intermittent fasting group (*n* = 9); *Differences within group, post *vs* pre (*p* < 0.05), by ANOVA two-way repeated measures mixed model, Sidak *post hoc*.

After 8 weeks of intervention with intermittent fasting and/or physical exercise, the IF + EX group showed a significant reduction in %body fat (*p* < 0.001), of approximately −4% (Δ%). The EX group also showed a significant loss in %body fat (*p* = 0.043), with a reduction of −2.3% (Δ%), slightly less expressive than the IF + EX-group. There were no differences between groups and the IF-group showed no changes. The study primary outcome of %fat-free mass also responded favorably to the intervention, since a significant increase was observed in the IF + EX-group (*p* < 0.001), of approximately +3.3% (Δ%), and in the EX-group (*p* = 0.043), of approximately +2% (Δ%), with no differences between groups. The IF group showed no changes (Figure 2).

### Food Consumption

**FIGURE 3:**
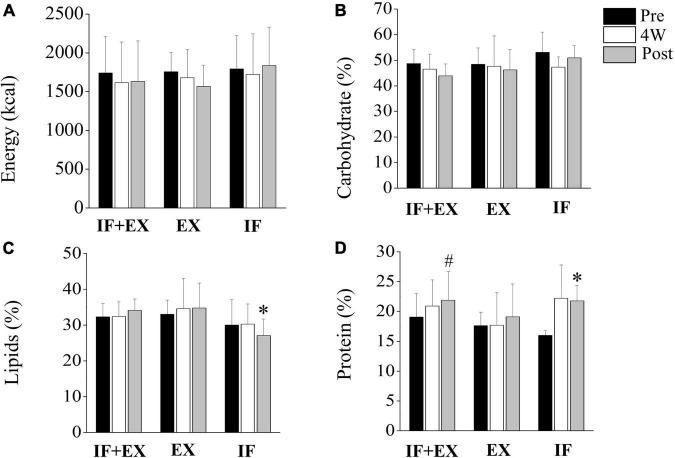
Food consumption pre- and post-intervention. IF + EX, intermittent fasting group in conjunction with exercise (*n* = 15); EX, exercise group (*n* = 11); IF, intermittent fasting group (*n* = 7); 4W, fourth week. Values expressed as mean ± standard deviation. **(A,B)**: no differences for energy (kcal) and carbohydrate (%) consumption. **(C)** *Difference between groups post intervention (IF *vs* IF + EX and EX); **(D)** *Difference within group post *vs* pre for IF group; ^#^difference within group post *vs* pre for IF + EX group, (*p* < 0.05), by ANOVA two-way mixed repeated measure, Sidak *post hoc*, and Welch’s correction for macronutrients.

Figure 3 illustrates the results of food consumption in calories and macronutrients. None of the groups showed differences in the consumption of carbohydrates and calories throughout the intervention. However, protein consumption increased in the IF + EX-group post-intervention compared to pre (*p* = 0.044) and the same occurred in the IF-group (*p* = 0.006), without differences between groups. The average protein intakes adjusted for body weight were 0.8 g/kg/day (pre) and 0.9 g/kg/day (post) in the IF + EX-group, 0.9 g/kg/day (pre) and 0.8 g/kg/day (post) for the EX-group, and 0.7 g/kg/day (pre) and 1.0 g/kg/day (post) for the IF-group. Lipid consumption significantly decreased in the IF-group post-intervention compared to the other groups at the same time (*p* = 0.011).

### Nitrogen Balance

**FIGURE 4:**
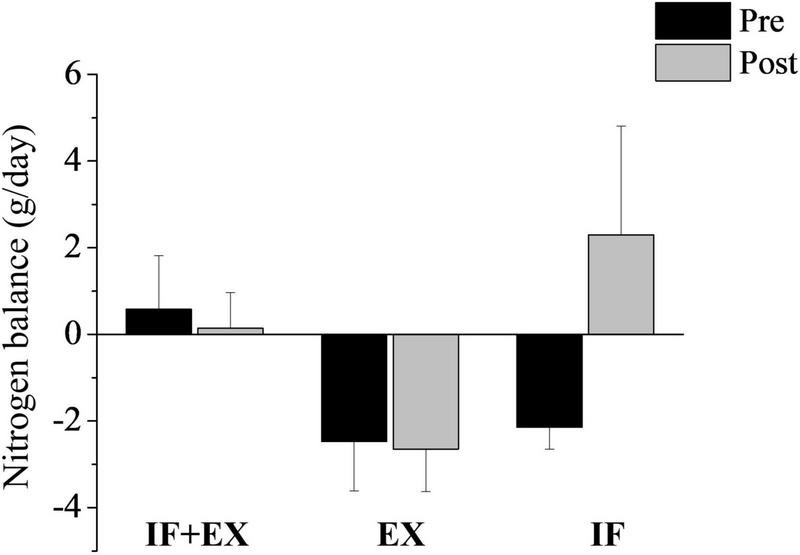
Nitrogen balance (g/day) pre- and post-intervention. IF + EX, intermittent fasting group in conjunction with exercise (*n* = 14); EX, exercise group (*n* = 11); IF, intermittent fasting group (*n* = 7). Values expressed as mean ± standard error. *Difference within the group, post vs pre, by ANOVA two-way mixed repeated measure, Sidak *post hoc*, and Welch’s correction.

The NB results showed that there were no changes in this parameter post-interventions and all groups maintained a neutral NB (range −4 to +4), as illustrated in Figure 4.

### High-Intensity Interval Training Improves Physical Fitness and Strength

Regarding the physical fitness variables shown in Table 4, all parameters improved significantly in the IF + EX and EX groups post-intervention, but there were no differences between groups. There was an improvement in the aerobic capacity of the participants, due to the increase observed in the time of execution of the maximum effort test (*p* < 0.001). Furthermore, the IF + EX group showed increase in VO_2_max post-intervention (*p* = 0.01), without no changes for the EX-group. There was no difference between groups at baseline (*p* = 0.75) and post-intervention (*p* = 0.13) for the VO_2_max variable. For muscle strength tests, the 1 RM leg 45° test improved 21% in the IF + EX -group and 29.1% in the EX-group (*p* < 0.001). In the bench press 1 RM test, the IF + EX-group presented improvements of 15.4%, while the EX-group improved 25.7% (*p* < 0.001). These results reflect an increased strength endurance capacity associated with the applied training protocol. Regarding the dynamometry tests, only the IF + EX-group responded significantly, showing an increase in strength performed post-intervention, both for the dorsal (*p* = 0.007) and handgrip tests (*p* = 0.001). The IF + EX and EX groups showed significant improvement in the test of maximum repetitions performed in 1 min for the abdominal, flexion, and squat exercises (*p* < 0.001), which represents a better adaptation to the effort. The HR_max_ of the EX-group significantly decreased post-intervention (*p* = 0.039), with no differences between groups.

**TABLE 4:** Physical fitness (aerobic and muscle strength) pre and post intervention.

	IF + EX (*n* = 15)			EX (*n* = 11)			
Variable	Pre	Post	Δ %	Pre	Post	Δ %	*p* Value
Shuttle Walking Test (min)	11 ± 0.8	12 ± 1*	8	11 ± 1	12 ± 1*	9	<0.001
Abdominal test (reps)	24 ± 3	35 ± 3*	48	27 ± 3	37 ± 1*	47	<0.001
Push-up test (reps)	20 ± 5	25 ± 4*	43	18 ± 2	24 ± 5*	30	<0.001
Squat test (reps)	28 ± 2	37 ± 3*	32	26 ± 2	36 ± 2*	37	<0.001
1RM Leg 45° test (kg)	253 ± 58	300 ± 67*	21	221 ± 46	279 ± 57*	29	<0.001
1RM Bench Press test (kg)	23 ± 8	26 ± 9*	15	20 ± 5	25 ± 7*	25	<0.001
Dorsal Dynamometer (kg/f)	84 ± 11	89 ± 13^#^	5	82 ± 10	85 ± 10	4	0.007
Handgrip (kg/f)	67 ± 8	73 ± 9^#^	10	67 ± 8	72 ± 13	6	0.001
HR_max_ (bpm/min)	183 ± 14	185 ± 12	1	187 ± 8	180 ± 9**	−4	0.039
VO_2_max (mL/kg/min)	30 ± 4	33 ± 2^#^	12	30 ± 4	32 ± 4	5	0.017

*1RM, 1 repetition of maximal strength; Reps, number of executed repetitions; HR_max_, Maximum heart rate; VO_2_max, maximum oxygen consumption; IF + EX, intermittent fasting in conjunction with exercise group; EX, exercise group; Δ%, variation between pre and post of each group. Values expressed as mean ± standard deviation. Symbols: Difference within groups (post vs pre), p < 0.05, by ANOVA two-way repeated measures mixed model, post hoc Sidak. ^#^Difference within group, by Wilcoxon test. Effect size: η^2^ = 0.51 (high for shuttle walking test).*

## Discussion

The main findings of the present study were: (a) weight loss after 8 weeks of IF combined with HIIT training; (b) IF combined with HIIT training promoted a greater gain in fat-free mass and greater loss of body fat than exercise in isolation, which was also able to improve body composition; (c) NB remained in equilibrium; and (d) HIIT improved aerobic fitness and muscle strength.

Stekovic et al. (38) in a prospective cohort study, found no significant differences in resting energy expenditure in individuals without obesity undergoing ADF for 4 weeks, but in the present investigation was observed that the IF alone, without the action of the exercise, reduced the RMR. The authors also observed improvement in the fat mass/lean mass ratio, as reported in our results, and in cardiovascular disease parameters. Recently, scientists revealed that an IF protocol involving a restricted diet within a 10-h window with 14 h of fasting in patients with metabolic syndrome and the absence of physical exercise, led to a significant reduction in body weight, which does not corroborate the results of the present work, because again, isolated IF did not affect changes in body composition or weight loss, but a significant improvement in waist circumference was observed, as in the present investigation (39).

Bhutani et al. (22) found that the effects of interventions of ADF and endurance exercise in individuals with obesity for 12 weeks were more promising when combined and not when evaluated in isolation. In this sense, the authors observed −6 kg weight loss in the group that combined IF with exercise, −1 kg in the exercise group, and −3 kg for the IF group. We did not find weight loss with IF and EX performed alone, only when together (IF + EX), besides better metabolic outcomes with IF acting synergistically with exercise. According to the authors the group that performed the IF combined with exercise lost more body fat (−5 kg) compared to individuals who underwent only the IF (−2 kg), but the endurance training was not as efficient in improving body composition as the HIIT training achieved in the present study, as Bhutani et al. (22) did not find a loss of body fat in the exercise group or a gain in lean mass in any of the groups evaluated.

The present study highlights encouraging outcomes regarding the fat-free mass gain observed in women with obesity undergoing IF associated with HIIT, and we draw attention to the importance of preserving fat-free mass and a better lifestyle when an intermittent fasting is linked to physical exercise. A recent review broadly contributes to understanding the effects of HIIT in increasing lean/fat free mass (40). Although no positive changes in body composition were observed with the isolated IF, the present intervention also did not lead to loss of fat-free mass, as observed in the clinical trial by Lowe et al. (41). The authors found a significant reduction in lean mass (calculated as fat-free mass minus bone mineral content) in subjects with overweight and obesity submitted to the Time-Restricted Eating protocol, a type of IF, for 12 weeks. In addition, they drew attention to the high proportion of lean mass loss, representing approximately 65% of observed weight loss, and that commonly the lean mass reduction during weight loss accounts for only 20–30% (42).

It has been reported that continuous caloric restriction or intermittent fasting strategies can affect energy metabolism, reducing the resting metabolic rate (43, 44), as observed in the IF group of the present study. Although the isolated IF did not result in weight loss or improvement in body composition, it is possible to state that the participants performed the proposed protocol considering that the RMR reduced by 8.1% (Δ) post-intervention. On the other hand, exercise played an important role in preserving energy expenditure, even under caloric restriction.

Regarding NB, all groups presented neutral NB, ranging between −4 and +4 (21) throughout the intervention, suggesting that the protein intake offered on fasting days associated with exercise was sufficient to maintain NB equilibrium and that the NB was also in agreement with the individuals’ usual caloric intake. The protein intake was in accordance with the recommendations according to the Dietary Reference Intakes - DRIs (0.8–1.0 g/kg/day) (25) corroborating with other authors (10) and added to the exercise efficiency, leading to a lower risk of fat free mass depletion. This is the first study to evaluate NB against an IF and HIIT protocol in women with obesity. The results of Campbell et al. (18), who evaluated the effect of continuous caloric restriction associated with resistance exercise in women with overweight or obesity for 16 weeks, corroborate our findings by observing neutral nitrogen balance during the caloric restriction process. Meckling and Sherfey (45) also evaluated the NB of women with obesity submitted to exercise and a low-calorie diet for 12 weeks and also found neutral NB and adequate protein intake.

The results of food consumption were favorable, as there was no caloric surplus in the *ad libitum* periods, since we did not observe changes in energy and carbohydrate intake and we still found significantly reduced lipid consumption after 8 weeks of IF in the absence of exercise. These outcomes are in line with other studies that evaluated reduced or increased hunger on unrestricted days without changes in energy intake (44, 46).

High-intensity interval training and the achievement of simultaneous gains in muscle mass and cardiorespiratory capacity is still not clear (40) and the present investigation contributes, from a metabolic point of view, to the obscurity of these aspects. Steckling et al. (47) in line with our findings, also considered HIIT training as an efficient strategy to improve cardiorespiratory fitness and functional parameters of individuals with obesity. Studies show that HIIT requires less time than conventional moderate-intensity exercise options to obtain health benefits including increased aerobic fitness which corroborates with the present study (48–50).

Adherence to a dietary intervention has been widely discussed in the context of obesity. There are several aspects that can influence adherence to a dietary protocol, such as food preferences, food availability, and, especially, the triggers that motivate the individual to maintain or change the diet. Undeniably, behavioral factors and environments also govern the degree of commitment of the individual. The fact is that strategies capable of improving adherence to diets are strongly linked to the success of these dietary therapies for weight loss, improvement in body composition, and health gains in general (51). In the present study, we observed a high number of dropouts in the three groups, but especially in the IF group, which means that there was low adherence to the proposed protocol. Adherence to IF is challenging and some aspects such as susceptibility to over-eating and possible adverse effects, have been associated with loss to follow-up or discontinued intervention in different IF protocols (50, 52).

In agreement with the present study, Bhutani et al. (22) and Jospe et al. (10) also found greater dropouts in the IF group and in the case of Bhutani et al. (22) when IF was combined with exercise, the authors also found that adherence was greater. Although the number of dropouts in the IF group was high, it is important to note that the loss to follow-up did not only occur due to the intervention, as there were other reasons. Intervention time is another point that deserves attention and is closely linked to motivational issues, which in obesity is something very complex. IF protocols applied for a long period, such as 12 months, tend to reduce adherence or stall the effects of weight loss (10), while interventions with shorter periods of time may affect diet adherence less, a fact that was not observed in the present 8-week intervention. In addition, as in the present study, other authors have also revealed physical exercise as an important motivational tool, even more so when linked to nutritional intervention, clearly superior to diet and exercise programs applied alone (22, 53), since we demonstrated the lowest dropout rates from the study in the group submitted to IF and exercise.

We emphasize that the original hypothesis of the present study contributes to the scientific community, which includes few clinical trials in less controlled living conditions, compared to the vast majority of experimental studies. Evidence on the long-term effectiveness of the possible synergism found between IF and HIIT is still minimal, reinforcing the idea that it is necessary to explore and better clarify these strategies in obesity.

### Study Limitations

The present study has limitations that deserve to be highlighted. We believe that performing a physical training protocol for a longer period may result in even better metabolic outcomes, since 8 weeks may be the beginning of post-adaptive physiological changes to exercise. In addition, the intermittent fasting protocol itself may have been limiting, as considering only 2 days a week of combined complete fasting with a restricted diet may not be enough to generate a caloric deficit representative for weight loss and improvements in body composition, considering the longer period of time with *ad libitum* eating. In addition, at baseline the EX group started the intervention with significantly lower body weight and BMI than the IF group, which means that these initial differences also represent a limitation of the study. Another limiting aspect was the encouragement given by the evaluators during the physical tests, despite all of them remaining blind to the treatments. The IF group did not perform the physical tests making it impossible for the outcomes of the physical capacities of this group to be compared with the other groups that performed the tests (IF + EX and EX) and in this aspect it is also a limitation. Still with regard to physical training, non-exercise activities were not monitored, but it is known that they commonly do not affect the outcomes of physical training. From the beginning, all participants were instructed not to perform any other structured exercise and the group submitted only to IF, to maintain a sedentary lifestyle throughout the intervention period.

We emphasize that the sample size reached at the end of the study deserves to be reported as another limitation.

## Conclusion

Combining IF with HIIT can be a good strategy for increasing fat-free mass, reducing body fat and weight, promoting nitrogen balance equilibrium, and improving physical fitness and strength in women with obesity. Additional studies are needed to further elucidate these outcomes using a larger sample size for a longer intervention time. The present study presents important contributions to the scientific community, since it is one of the few studies with IF and HIIT in humans showing protection against the decline in fat-free mass, the first to evaluate the nitrogen balance under these intervention conditions, and also for promoting a non-sedentary lifestyle. Furthermore, our outcomes may represent potential targets for the management of obesity, a serious public health problem that deserves attention.

## Data Availability Statement

The original contributions presented in the study are included in the article/Supplementary Material, further inquiries can be directed to the corresponding author.

## Ethics Statement

The studies involving human participants were reviewed and approved by the Ethics Committee of the School of Physical Education and Sport of Ribeirão Preto (EEFERP-USP) and of the University Hospital of the Faculty of Medicine of Ribeirão Preto approved the study (nos. 13359319.3.0000.5659 and 13359319.3.3001.5440, respectively). The patients/participants provided their written informed consent to participate in this study.

## Author Contributions

GB and EF contributed to conception and design of the study. GB, EVF, and CB organized the database. GB, EVF, JN, CB, JM, and EF contributed to the methodology. GB and EVF performed the statistical analysis. GB, EVF, and EF wrote the first draft of the manuscript. GB, EVF, GA, and GO wrote sections of the manuscript. EF performed the funding acquisition. All authors contributed to manuscript revision, read, and approved the submitted version.

## Conflict of Interest

The authors declare that the research was conducted in the absence of any commercial or financial relationships that could be construed as a potential conflict of interest.

## Publisher’s Note

All claims expressed in this article are solely those of the authors and do not necessarily represent those of their affiliated organizations, or those of the publisher, the editors and the reviewers. Any product that may be evaluated in this article, or claim that may be made by its manufacturer, is not guaranteed or endorsed by the publisher.

## Supplementary Material

Supplementary Material 1 Nutrient composition of two meals the intermittent fasting protocol.

Supplementary Material 2 Supplementary material checklist Consort.

## References

[1] Chooi YC Ding C Magkos F. The epidemiology of obesity. *Metabolism.* (2019). 92:6–10. 10.1016/j.metabol.2018.09.005 30253139

[2] Lim SS Vos T Flaxman AD Danaei G Shibuya K Adair-Rohani H A comparative risk assessment of burden of disease and injury attributable to 67 risk factors and risk factor clusters in 21 regions, 1990-2010: a systematic analysis for the global burden of disease study 2010. *Lancet.* (2012) 380:2224–60. 10.1016/S0140-6736(12)61766-8 23245609 PMC4156511

[3] World Health Organization. *Obesity and Overweight.* (2022). Available online at: http://www.who.int/mediacentre/factsheets/fs311/en/ (accessed January 26, 2022).

[4] Goossens GH. The role of adipose tissue dysfunction in the pathogenesis of obesity-related insulin resistance. *Physiol Behav.* (2008) 94:206–18. 10.1016/j.physbeh.2007.10.010 18037457

[5] Harris L Hamilton S Azevedo LB Olajide J De Brún C Waller G Intermittent fasting interventions for treatment of overweight and obesity in adults: a systematic review and meta-analysis. *JBI Database Syst Rev Implement Rep.* (2018) 16:507–47. 10.11124/JBISRIR-2016-003248 29419624

[6] Cienfuegos S Gabel K Kalam F Ezpeleta M Wiseman E Pavlou V Effects of 4- and 6-h time-restricted feeding on weight and cardiometabolic health: a randomized controlled trial in adults with obesity. *Cell Metab.* (2020) 32:366–78.e3. 10.1016/j.cmet.2020.06.018 32673591 PMC9407646

[7] Tinsley GM La Bounty PM. Effects of intermittent fasting on body composition and clinical health markers in humans. *Nutr Rev.* (2015) 73:661–74. 10.1093/nutrit/nuv041 26374764

[8] Wewege M van den Berg R Ward RE Keech A. The effects of high-intensity interval training vs. moderate-intensity continuous training on body composition in overweight and obese adults: a systematic review and meta-analysis. *Obes Rev.* (2017) 18:635–46. 10.1111/obr.12532 28401638

[9] Catenacci VA Pan Z Ostendorf D Brannon S Gozansky WS Mattson MP A randomized pilot study comparing zero-calorie alternate-day fasting to daily caloric restriction in adults with obesity. *Obesity (Silver Spring).* (2016) 24:1874–83. 10.1002/oby.21581 27569118 PMC5042570

[10] Jospe MR Roy M Brown RC Haszard JJ Meredith-Jones K Fangupo LJ Intermittent fasting, paleolithic, or Mediterranean diets in the real world: exploratory secondary analyses of a weight-loss trial that included choice of diet and exercise. *Am J Clin Nutr.* (2020) 111:503–14. 10.1093/ajcn/nqz330 31879752

[11] Cho AR Moon JY Kim S An KY Oh M Jeon JY Effects of alternate day fasting and exercise on cholesterol metabolism in overweight or obese adults: a pilot randomized controlled trial. *Metabolism.* (2019) 93:52–60. 10.1016/j.metabol.2019.01.002 30615947

[12] McCarthy D Berg A. Weight loss strategies and the risk of skeletal muscle mass loss. *Nutrients.* (2021) 13:2473. 10.3390/nu13072473 34371981 PMC8308821

[13] Weiss EP Jordan RC Frese EM Albert SG Villareal DT. Effects of weight loss on lean mass, strength, bone, and aerobic capacity. *Med Sci Sports Exerc.* (2017) 49:206–17. 10.1249/MSS.0000000000001074 27580151 PMC5161655

[14] Enríquez Guerrero A San Mauro Martín I Garicano Vilar E Camina Martín A. Effectiveness of an intermittent fasting diet versus continuous energy restriction on anthropometric measurements, body composition and lipid profile in overweight and obese adults: a meta-analysis. *Eur J Clin Nutr.* (2021) 75:1024–39. 10.1038/s41430-020-00821-1 33293678

[15] Kalam F Gabel K Cienfuegos S Wiseman E Ezpeleta M Steward M Alternate day fasting combined with a low-carbohydrate diet for weight loss, weight maintenance, and metabolic disease risk reduction. *Obes Sci Pract.* (2019) 5:531–9. 10.1002/osp4.367 31890243 PMC6934424

[16] Varady KA. Intermittent versus daily calorie restriction: which diet regimen is more effective for weight loss? *Obes Rev.* (2011) 12:593–601. 10.1111/j.1467-789X.2011.00873.x 21410865

[17] Patikorn C Roubal K Veettil SK Chandran V Pham T Lee YY Intermittent fasting and obesity-related health outcomes an umbrella review of meta-analyses of randomized clinical trials. *JAMA Netw Open.* (2021) 4:e2139558. 10.1001/jamanetworkopen.2021.39558 34919135 PMC8683964

[18] Campbell WW Haub MD Wolfe RR Ferrando AA Sullivan DH Apolzan JW Resistance training preserves fat-free mass without impacting changes in protein metabolism after weight loss in older women. *Obesity.* (2009) 17:1332–9. 10.1038/oby.2009.2 19247271 PMC4299870

[19] Sperlich B Wallmann-Sperlich B Zinner C Von Stauffenberg V Losert H Holmberg HC. Functional high-intensity circuit training improves body composition, peak oxygen uptake, strength, and alters certain dimensions of quality of life in overweight women. *Front Physiol.* (2017) 8:172. 10.3389/fphys.2017.00172 28420999 PMC5376588

[20] Motta V. *Bioquímica Clínica Para o Laboratório: Princípios e Interpretações.* 4^2^ ed. São Paulo: Médica Missau (2003).

[21] Dickerson RN. Nitrogen balance and protein requirements for critically Ill older patients. *Nutrients.* (2016) 8:226. 10.3390/nu8040226 27096868 PMC4848694

[22] Bhutani S Klempel MC Kroeger CM Trepanowski JF Varady KA. Alternate day fasting and endurance exercise combine to reduce body weight and favorably alter plasma lipids in obese humans. *Obesity.* (2013) 21:1370–9. 10.1002/oby.20353 23408502

[23] Westerterp-Plantenga MS Lemmens SG Westerterp KR. Dietary protein – its role in satiety, energetics, weight loss and health. *Br J Nutr.* (2012) 108:S105–12. 10.1017/S0007114512002589 23107521

[24] Patterson RE Sears DD. Metabolic effects of intermittent fasting. *Annu Rev Nutr.* (2017) 37:371–93. 10.1146/annurev-nutr-071816-064634 28715993 PMC13170603

[25] Institute of medicine. *Dietary Reference Intakes for Energy, Carbohydrate, Fiber, Fat, Fatty Acids, Cholesterol, Protein and Amino Acids.* Washington, DC: The National Academies Press (2005). 10.17226/10490

[26] Foster C Florhaug JA Franklin J Gottschall L Hrovatin LA Parker S A new approach to monitoring exercise training. *J Strength Cond Res.* (2001) 15:109–15. 11708692

[27] Rossi L Caruso L Galante AP. *Avaliação Nutricional: Novas Perspectivas.* São Paulo: Roca (2009).

[28] Albersen M Bonthuis M De Roos NM Van Den Hurk DAM Carbasius-Weber E Hendriks MMWB Whole body composition analysis by the BodPod air-displacement plethysmography method in children with Phenylketonuria shows a higher body fat percentage. *J Inherit Metab Dis.* (2010) 33:S283–8. 10.1007/s10545-010-9149-8 20574715 PMC3757265

[29] Ferrannini E. The theoretical bases of indirect calorimetry: a review. *Metabolism.* (1988) 37:287–301. 10.1016/0026-0495(88)90110-2 3278194

[30] Weir JBDB. New methods for calculating metabolic rate with special reference to protein metabolism. *J. Physiol.* (1949) 109:1–9. 10.1113/jphysiol.1949.sp004363 15394301 PMC1392602

[31] Frayn K. Calculation of substrate oxidation rates in vivo from gaseous exchange. *J Appl Physiol.* (1983) 55:628–34. 10.1152/jappl.1983.55.2.628 6618956

[32] Friedewald WT Levy RI Fredrickson DS. Estimation of the concentration of low-density lipoprotein cholesterol in plasma, without use of the preparative ultracentrifuge. *Clin Chem.* (1972) 18:499–502. 4337382

[33] Grimble GK West MF Acuti ABC Rees RG Hunjan MK Webster JD Assessment of an automated chemiluminescence nitrogen analyzer for routine use in clinical nutrition. *J Parenter Enter Nutr.* (1988) 12:100–6. 10.1177/0148607188012001100 3125351

[34] Singh SJ Morgan MDL Scott S Walters D Hardman AE. Development of a shuttle walking test of disability in patients with chronic airways obstruction. *Thorax.* (1992) 47:1019–24. 10.1136/thx.47.12.1019 1494764 PMC1021093

[35] Lima LP Leite HR Matos MA Neves CC Lage VS Silva GD Cardiorespiratory fitness assessment and prediction of peak oxygen consumption by incremental shuttle walking test in healthy women. *PLoS One.* (2019) 14:e0211327. 10.1371/journal.pone.0211327 30730949 PMC6366724

[36] Brzycki M. Strength testing—predicting a one-rep max from reps-to-fatigue. *J Phys Educ Recreat Danc.* (1993) 64:88–90. 10.1080/07303084.1993.10606684

[37] Dong B Wang Z Arnold L Song Y Wang HJ Ma J. The association between blood pressure and grip strength in adolescents: does body mass index matter. *Hypertens Res.* (2016) 39:919–25. 10.1038/hr.2016.84 27383511

[38] Stekovic S Hofer SJ Tripolt N Aon MA Royer P Pein L Alternate day fasting improves physiological and molecular markers of aging in healthy, non-obese humans. *Cell Metab.* (2019) 30:462–76.e5. 10.1016/j.cmet.2019.07.016 31471173

[39] Wilkinson MJ Manoogian ENC Zadourian A Lo H Fakhouri S Shoghi A Ten-hour time-restricted eating reduces weight, blood pressure, and atherogenic lipids in patients with metabolic syndrome. *Cell Metab.* (2020) 31:92–104.e5. 10.1016/j.cmet.2019.11.004 31813824 PMC6953486

[40] Callahan MJ Parr EB Hawley JA Camera DM. Can high-intensity interval training promote skeletal muscle anabolism? *Sport Med.* (2021) 51:405–21. 10.1007/s40279-020-01397-3 33512698

[41] Lowe DA Wu N Rohdin-Bibby L Moore AH Kelly N Liu YE Effects of time-restricted eating on weight loss and other metabolic parameters in women and men with overweight and obesity: the TREAT randomized clinical trial. *JAMA Intern Med.* (2020) 180:1491–9. 10.1001/jamainternmed.2020.4153 32986097 PMC7522780

[42] Magkos F Fraterrigo G Yoshino J Luecking C Kirbach K Kelly SC Effects of moderate and subsequent progressive weight loss on metabolic function and adipose tissue biology in humans with obesity. *Cell Metab.* (2016) 23:591–601. 10.1016/j.cmet.2016.02.005 26916363 PMC4833627

[43] Coutinho SR Halset EH Gåsbakk S Rehfeld JF Kulseng B Truby H Compensatory mechanisms activated with intermittent energy restriction: a randomized control trial. *Clin Nutr.* (2018) 37:815–23. 10.1016/j.clnu.2017.04.002 28446382

[44] Hoddy KK Marlatt KL Çetinkaya H Ravussin E. Intermittent fasting and metabolic health: from religious fast to time-restricted feeding. *Obesity.* (2020) 28:S29–37. 10.1002/oby.22829 32700827 PMC7419159

[45] Meckling KA Sherfey R. A randomized trial of a hypocaloric high-protein diet, with and without exercise, on weight loss, fitness, and markers of the metabolic syndrome in overweight and obese women. *Appl Physiol Nutr Metab.* (2007) 32:743–52. 10.1139/H07-059 17622289

[46] Harvey J Howell A Morris J Harvie M. Intermittent energy restriction for weight loss: spontaneous reduction of energy intake on unrestricted days. *Food Sci Nutr.* (2018) 6:674–80. 10.1002/fsn3.586 29876119 PMC5980333

[47] Steckling FM Farinha JB Figueiredo FC Santos DL Dos Bresciani G Kretzmann NA High-intensity interval training improves inflammatory and adipokine profiles in postmenopausal women with metabolic syndrome. *Arch Physiol Biochem.* (2019) 125:85–91. 10.1080/13813455.2018.1437750 29431478

[48] Gillen JB Gibala MJ. Is high-intensity interval training a time-efficient exercise strategy to improve health and fitness? *Appl Physiol Nutr Metab.* (2014) 39:409–12. 10.1139/apnm-2013-0187 24552392

[49] Sloth M Sloth D Overgaard K Dalgas U. Effects of sprint interval training on VO2max and aerobic exercise performance: a systematic review and meta-analysis. *Scand J Med Sci Sport.* (2013) 23:341–52. 10.1111/sms.12092 23889316

[50] Milanoviæ Z Sporiš G Weston M. Effectiveness of high-intensity interval training (HIT) and continuous endurance training for VO2max improvements: a systematic review and meta-analysis of controlled trials. *Sport Med.* (2015) 45:1469–81. 10.1007/s40279-015-0365-0 26243014

[51] Freire R. Scientific evidence of diets for weight loss: different macronutrient composition, intermittent fasting, and popular diets. *Nutrition.* (2020) 69:110549. 10.1016/j.nut.2019.07.001 31525701

[52] Trepanowski JF Kroeger CM Barnosky A Klempel MC Bhutani S Hoddy KK Effect of alternate-day fasting on weight loss, weight maintenance, and cardioprotection among metabolically healthy obese adults: a randomized clinical trial. *JAMA Intern Med.* (2017) 177:930–8. 10.1001/jamainternmed.2017.0936 28459931 PMC5680777

[53] Lemstra M Bird Y Nwankwo C Rogers M Moraros J. Weight loss intervention adherence and factors promoting adherence: a meta-analysis. *Patient Prefer Adherence.* (2016) 10:1547–59. 10.2147/PPA.S103649 27574404 PMC4990387

