# Supplementary material for: Impact of Intermittent Fasting Combined With High-Intensity Interval Training on Body Composition, Metabolic Biomarkers, and Physical Fitness in Women With Obesity
Source: Front Nutr. 2022 May 26;9:884305. doi: 10.3389/fnut.2022.884305 (PMC9178202; doi:10.3389/fnut.2022.884305)
Supplement: Supplementary Material 1 — Nutrient composition of two meals the intermittent fasting protocol. [file Data_Sheet_1.pdf]

Supplementary Material 1

Table 1: Nutrient composition of two meals the intermittent fasting protocol.

	Meal 1	Meal 2	
**Foods**	Whole bread (50g)	Whole pasta (110g)	
	Shredded chicken breast (60g)	Ground beef (75g)	
	Tomato(24g)	Tomato sauce (75g)	
	Ricotta cheese (70g)		
**Nutrients**			**Daily total**
Energy (kcal)	326	330	656
Protein (g)	32,68 (40%)	33,82 (40%)	66,50 (40%)
Carbohydrate (g)	28,37 (34%)	34,98 (42%)	63,35 (38%)
Fat (g)	9,44 (26%)	6,76 (18%)	16,20 (22%)

Note: There were no restrictions on the consumption of green leafy salad.
