# Supplementary figures and images for: Impact of Intermittent Fasting Combined With High-Intensity Interval Training on Body Composition, Metabolic Biomarkers, and Physical Fitness in Women With Obesity
Source: Front Nutr. 2022 May 26;9:884305. doi: 10.3389/fnut.2022.884305 (PMC9178202; doi:10.3389/fnut.2022.884305)

## CONSORT 2010 Flow Diagram

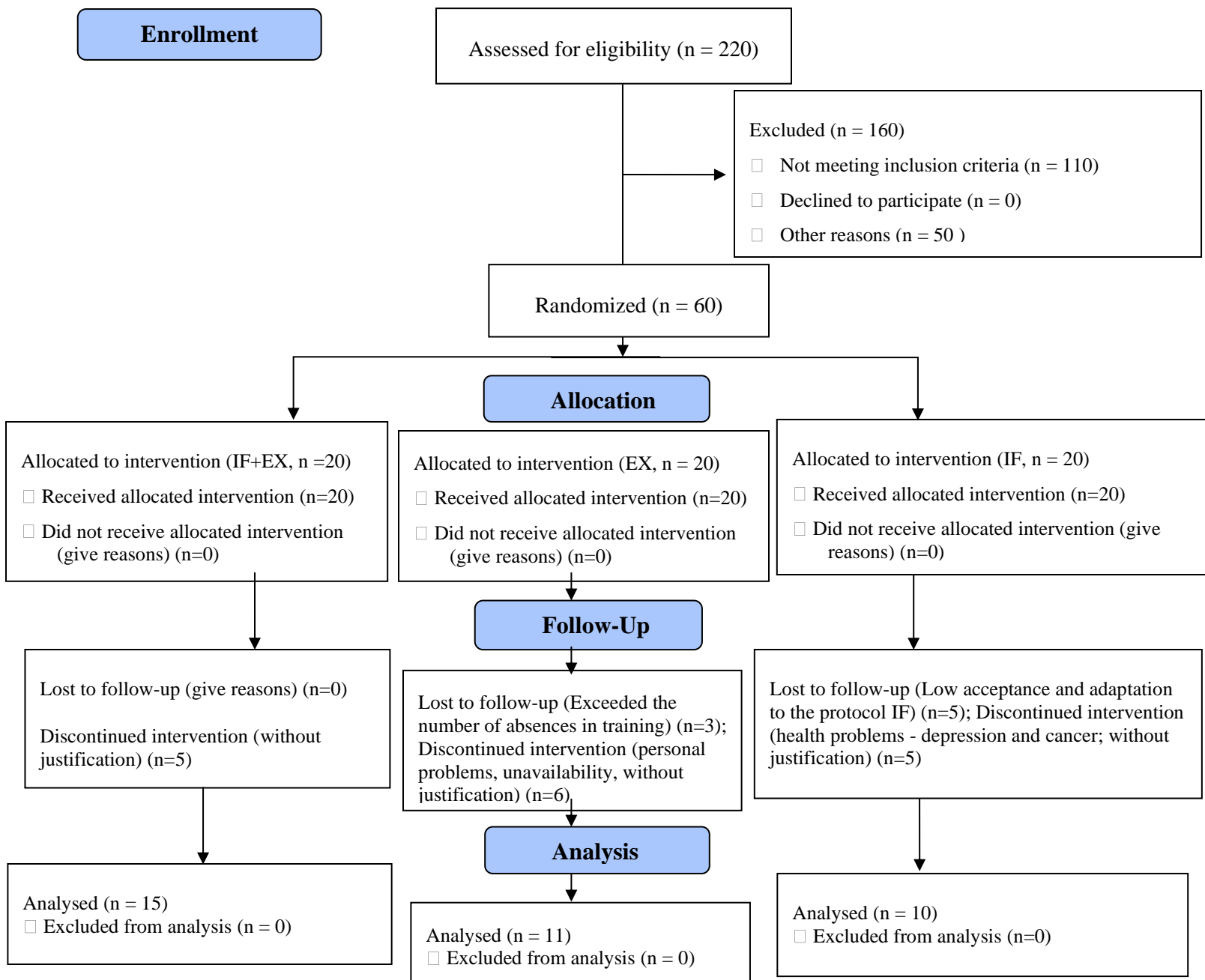

Supplement: Supplementary Material 2 — Supplementary material checklist Consort. [file Data_Sheet_2.pdf]
